# Observations on the Analysis of Urinary Calculi, Particularly Those of a Mixed Nature

**Published:** 1827-01

**Authors:** John F. Wood

**Affiliations:** Lecturer on Chemistry in the School in Great Windmill-street.


### URINARY CALCULI.

*Observations on the Analysis of Urinary Calculi, particularly those of a mixed nature.* By John F. Wood, Lecturer on Chemistry in the School in Great Windmill-street.

About two years ago, an opportunity occurred to me of analysing the urinary calculi contained in the museum in Great Windmill-street; and since that time I have also been favoured by Mr. Cross, of Norwich, and by the medical officers of the Kent and Canterbury Hospital, with the use of their collections for the same purpose. The number of these concretions thus brought under my notice having been considerable, I have ventured to publish the results of their analysis, in the hope of at least affording some assistance to any one who may hereafter be inclined to prosecute inquiries into this very interesting and important branch of medical chemistry.

It will be at once perceived that I have avoided entering into the mechanical and chemical qualities of urinary calculi with any degree of minuteness, as sufficiently accurate descriptions of them are to be found in most chemical works, particularly in the very excellent publication of the late Dr. Marcet upon this subject. I have therefore confined myself to a detail of the most ready means of distinguishing them from one another, and of analysing them when the various ingredients are intimately blended together. Indeed, I know of no publication containing any directions for the analysis of mixed calculi; and the want of such information proved at the commencement a source of considerable inconvenience to me, from which I hope in some degree to relieve others, by a detail of the means employed for its removal.

Calculi, in order to be analysed, must first be divided, that the different laminæ may be seen, and analysed apart from the others. This operation should be carefully performed with a very fine saw, in that direction which will expose the most extensive surface when divided: a few grains should then be scraped with a penknife from each lamina, commencing with the nucleus, and proceeding towards the exterior; care being taken to select the central portion of each layer, that it may not be mixed with those on either side of it.

The apparatus required is simple, and the materials are few in number. Some watch-glasses, a spirit-lamp, a brass stand with rings for holding the glasses, glass rods for stirring the solutions,* a pair of platina forceps, a piece of platina foil,

* I have found small glass tubes, drawn out to a fine point, which may be readily done by the heat of a spirit-lamp, extremely useful for removing the fluid from a blowpipe, and some stoppered bottles, containing pure nitric, muriatic, and acetic acids, solutions of potassa, ammonia, carbonate of ammonia, and oxalate of ammonia, with distilled water, complete the list.*

At present only six ingredients have been discovered in urinary calculi,—viz. uric acid, urate of ammonia, cystic oxide, phosphate of lime, oxalate of lime, and the phosphate of ammonia and magnesia. One or two other substances have been discovered, but in so few instances as not to deserve mention with the others: such are carbonate of lime, and the two substances discovered by Dr. Marcet, the xanthic oxide and the fibrinous calculus.

These may be distinguished from each other by the following tests:—Three of them are soluble in cold solution of potassa,—the uric acid, urate of ammonia, and cystic oxide. The first dissolves in cold solution of potassa, and evolves no ammoniacal odour during its solution. On the addition of dilute muriatic acid, a copious white precipitate falls.

Urate of ammonia resembles the last, excepting that copious fumes of ammonia are disengaged during the solution, which may be detected simply by the smell, or by holding the stopper of the nitric or muriatic acid bottle over the glass containing the materials, when dense white clouds of nitrate or muriate of ammonia will instantly be produced; or a piece of moistened turmeric paper will be reddened, if held over the glass. In either case the effect will be much increased by gently heating the glass. This variety is also soluble, with comparative ease, in distilled water; a fact first noticed by Dr. Prout.

Cystic oxide calculi dissolve readily in cold solution of potassa, give off no ammoniacal fumes, and afford no precipitate on the addition of dilute muriatic acid.

Four varieties are soluble in dilute acids,—namely, the phosphate of lime, the phosphate of ammonia and magnesia, or triple phosphate, the oxalate of lime, and the cystic oxide; and, of course, any mixture of them. All these are soluble in muriatic acid diluted with four parts of water, and are all precipitated by ammonia and the fixed alkalies, excepting the cystic oxide, which, being equally soluble in acids the undissolved portion, which is easily effected by carefully immersing the point in the fluid, and applying the mouth to the other extremity. They are also useful for experiments on very minute quantities; as, by filling them with any fluid, and closing the larger end with the finger, the fluid is retained, and may be allowed to escape gradually in quantities as small as can possibly be required.

* Pieces of broken window-glass answer for many purposes as well as watch-glasses, and are of course much more economical.

and alkalies, is of course not affected. It may, however, be thrown down by carbonate of ammonia. These may be distinguished from each other in the following manner:

The phosphate of lime calculus, when heated before the blowpipe, undergoes a very trifling alteration; no peculiar smell is perceived, excepting that of burning animal matter when the heat is first applied; the ash is white, not alkaline, dissolves readily and quickly in dilute acids; and the solution gives a precipitate with pure ammonia, and with its oxalate, provided there be no great excess of acid.

The triple phosphate likewise suffers very little alteration before the blowpipe, unless the heat be very strongly urged, when it fuses imperfectly. Copious fumes of ammonia are disengaged. The ash is generally brown, not alkaline; is soluble, but not readily, in dilute muriatic acid, and precipitable in the form of crystalline grains by ammonia, showing the re-formation of the triple phosphate. The oxalate of ammonia does not precipitate this salt when no phosphate of lime exists in combination with it.

The peculiar fetid odour disengaged on the application of heat, and its ready solubility in both acids and alkalies, sufficiently characterise the cystic oxide.

The oxalate of lime calculus, when heated before the blowpipe, blackens, is enlarged in volume, and leaves a great quantity of ash of an intense whiteness, which is soluble with effervescence in dilute muriatic acid, unless the heat have been intense and applied for a long time, when it dissolves quietly. Pure ammonia produces no precipitate from the solution, but a copious bulky one is thrown down by the oxalate of ammonia. This ash gives a deep red tinge to moistened turmeric paper, and is in fact pure lime.

Thus far the analysis is sufficiently easy, the calculi or laminæ consisting of only one ingredient: few calculi, however, are of a nature so simple, the greater number being composed of two or more ingredients, sometimes in separate laminæ, sometimes blended together; and it is in the examination of these that the greatest difficulty is experienced, which I shall endeavour, by the following directions, in some measure to remove.

Uric Acid and Oxalate of Lime.—Calculi of this composition, when digested in solution of potassa, washed and filtered, the potassa will dissolve all the uric acid, which may be obtained by adding dilute muriatic acid to the filtered liquor; and the solid residue, being heated before the blowpipe, will give the results mentioned above. This mixture generally decrepitates loudly when first heated.

Uric Acid and Phosphate of Lime.—This mixture may be treated as the last; the uric acid being dissolved out by potassa; and the remainder may either be heated before the blowpipe, or dissolved in dilute muriatic acid, and will afford the results mentioned under the head of phosphate of lime calculi.

Uric Acid and Triple Phosphate.—When calculi of this composition are digested in solution of potassa, the acid is separated; a very powerful ammoniacal odour is evolved; and the undissolved portion, when well washed and dissolved in dilute muriatic acid, will, on the addition of ammonia, be reconverted into triple phosphate, in the form of small acicular crystals.

Phosphate of Lime and Phosphate of Ammonia and Magnesia, or the Fusible Calculus.—This variety is immediately recognised by readily fusing before the blowpipe into an opake, white, shining enamel. It suffers very little diminution in bulk, gives off copious fumes of ammonia, and the ash is not alkaline.

Phosphate of Lime and Oxalate of Lime.—These calculi do not fuse before the blowpipe, but swell up in proportion to the quantity of oxalate of lime they contain; give out no smell of ammonia; the ash is alkaline, and, if it be dissolved in dilute muriatic acid, the solution precipitated by ammonia and filtered, a precipitate* is produced in the filtered fluid by the oxalate of ammonia. Phosphoric acid will dissolve out the phosphate, and leave the oxalate.†

Uric Acid and Fusible.—These calculi blacken before the blowpipe, diminish in bulk in proportion to the quantity of uric acid, evolve a powerful smell of ammonia, and presently fuse. The ash is very slightly alkaline, dissolves without effervescence in dilute muriatic acid, and is precipitable by ammonia. We are indebted to Dr. WOLLASTON for the following method of analysing calculi of this description:—Digest in cold distilled vinegar, which takes up only the triple phosphate; dilute muriatic acid will next separate the phosphate of lime; and the remainder, which will be the uric acid, may be recognised by the usual tests.

* This precipitate by oxalate of ammonia, after the pure alkali has produced its full effect, depends upon the existence of lime in the calculus, in combination with an acid easily separable by heat, leaving the lime in combination with the acid in which the ash is dissolved. From this combination ammonia will not detach the lime: it therefore passes the filter in solution, and is afterwards precipitated by the oxalate of ammonia. Now the only two acids of this kind hitherto found in calculi are the oxalic and the carbonic: if it be the latter, the calculus will dissolve, with effervescence, in any acid; if the former, the solution will proceed quietly.

† Dr. Wollaston.

Triple Phosphate and Oxalate of Lime.—This mixture does not fuse before the blowpipe, gives off a pungent ammoniacal odour, swells up in proportion to the quantity of the oxalate, and does not perceptibly diminish in quantity. The ash is more or less alkaline according to circumstances, dissolves readily in dilute muriatic acid, and, after ammonia has precipitated all it can throw down, a further deposition takes place in the filtered liquor on the addition of the oxalate of ammonia.

Fusible and Oxalate of Lime.—This calculus differs from the last only in undergoing a partial fusion after the heat has been applied a short time.

Many calculi contain uric acid in so small a proportion as not to influence their external character and appearance: in these it may readily be detected by placing a small portion of the calculus, or lamina, in powder, on a piece of glass, and adding a drop or two of pure nitric acid; heating it over a lamp, very carefully to prevent it from charring. Should there be the smallest quantity of uric acid, a red colour will appear as the mixture becomes dry, and will be deep in proportion to the quantity of uric acid. A drop or two of a solution of pure ammonia, added when it has cooled, will develope a beautiful purple tinge. If it be an object to ascertain the exact proportion of uric acid, take any given weight of the calculus, and digest it in dilute muriatic acid, and the undissolved portion will be the uric acid.

During my examination of the calculi in the museum of Great Windmill-street, I found one possessing some very extraordinary properties, resembling those attributed to the cystic oxide. Dr. Wollaston, the discoverer of this species of calculus, was so obliging as to sacrifice a portion of his own specimen, to afford comparative experiments; and these fully confirmed the identity of composition of the two calculi. In the same collection is another specimen of this species of concretion, which is extremely rare; for, amongst the many hundred calculi which have been analysed, only seven of this kind have hitherto been discovered, so that, with the addition of the two in this collection, there are but nine cystic oxide calculi at present on record.

In the accompanying table, I have arranged the calculi in a manner similar to that adopted by Dr. Prout, in his book on Calculous Diseases; and I may remark, once for all, that each specimen was divided, and each layer separately analysed, so that all confusion is avoided, and the result may be depended on as the real composition of the several calculi.

| General Character. | Particular Species. | Windmill-Street. | | Mr. Cross. | | Canterbury. | | Particular Total. | General Total. |
|---|---|---|---|---|---|---|---|---|---|
| URIC ACID | *Nearly pure | 25 | 29 | 13 | 17 | 7† | 10 | 45 | 56 |
| | Mixed intimately with a little oxalate of lime | 3 | | 3 | | — | | 6 | |
| | Urate of ammonia | 1 | | 1 | | 3 | | 5 | |
| CYSTIC OXIDE | | 2 | 2 | — | — | — | — | 2 | 2 |
| OXALATE OF LIME | Mulberry | 2 | 5 | 1 | 1 | 1 | 1 | 4 | 7 |
| | Hemp-seed calculus | 1‡ | | — | | — | | 1 | |
| | Containing a little uric acid | 1 | | — | | — | | 1 | |
| | With traces of uric acid and of phosphate of lime | 1 | | — | | — | | 1 | |
| PHOSPHATES | Phosphate of lime (nearly pure) | 2 | 20 | — | 1 | — | 1 | 2 | 22 |
| | Phosphate of lime, with a little oxalate of lime | 1 | | — | | — | | 1 | |
| | Phosphate of lime, with a very little carbonate of lime | 1 | | — | | — | | 1 | |
| | Triple phosphate | 1 | | — | | — | | 1 | |
| | Triple phosphate, with a little oxalate of lime | 1 | | — | | — | | 1 | |
| | Fusible calculus (pure) | 5 | | — | | — | | 5 | |
| | Fusible, with a little uric acid | 7 | | 1 | | 1 | | 9 | |
| | Phosphate of lime, succeeded by fusible | 2 | | — | | — | | 2 | |
| | Uric acid, and oxalate of lime | 1 | | 4 | | — | | 5 | |
| | Oxalate, and uric acid | 3 | | 1 | | 1 | | 5 | |
| | Uric acid, and oxalate of lime and uric acid mixed | 6 | | — | | 2 | | 8 | |
| | Uric acid, and phosphates | 4 | | 5 | | 3 | | 12 | |
| | Oxalate, and phosphates | 1 | | — | | — | | 1 | |
| | Oxalate, and phosphates containing a little uric acid | 4 | | — | | — | | 4 | |
| | Oxalate of lime containing a little uric acid, and phosphates | — | | 1 | | — | | 1 | |
| | Triple phosphate, and oxalate | 1 | | — | | — | | 1 | |
| | Uric acid, and fusible with uric acid | 2 | | — | | 2 | | 4 | |
| | Uric acid, and oxalate and phosphate of lime mixed | — | | — | | 1 | | 1 | |
| | Uric acid, and oxalate of lime and fusible mixed | — | | — | | [illegible]1 | | 1 | |
| | Oxalate of lime, and uric acid and phosphates mixed | 1 | | — | | 1 | | 2 | |

| | | | | | | | | | |
|---|---|---|---|---|---|---|---|---|---|
| ALTERNATING CALCULI | Oxalate of lime and uric acid, and oxalate of lime and triple phosphate | — | | — | | 1 | | 1 | |
| | Fusible with a little uric acid, and oxalate of lime | — | 26 | — | 16 | 1 | 18 | 1 | 60 |
| | Uric acid and oxalate of lime with a little triple phosphate, and oxalate and triple phosphate | — | | — | | 1 | | 1 | |
| | Uric acid, fusible and uric acid, and pure fusible | 1 | | — | | — | | 1 | |
| | Uric acid, oxalate, and phosphate | — | | 3 | | 1 | | 4 | |
| | Uric acid, urate of ammonia, and fusible | — | | — | | 1 | | 1 | |
| | Oxalate, fusible, and oxalate | — | | — | | 1 | | 1 | |
| | Oxalate and uric acid, uric acid, and fusible | — | | — | | 1 | | 1 | |
| | Urate of ammonia and oxalate of lime, fusible and uric acid | — | | 1 | | — | | 1 | |
| | Oxalate and uric acid, phosphate of lime (pure), and uric acid and phosphate of lime | — | | 1 | | — | | 1 | |
| | Oxalate of lime and uric acid, oxalate and fusible, fusible containing a very little oxalate, and pure fusible, (4 distinct layers) | 1 | | — | | — | | 1 | |
| | Uric acid, phosphate of lime, oxalate of lime, and triple phosphate, (4 distinct layers) | 1 | | — | | — | | 1 | |
| MIXED CALCULI | Uric acid and oxalate of lime | 1 | | 1 | | 3 | | 5 | |
| | Uric acid and fusible | 6§ | | — | | — | | 6 | |
| | Uric acid and urate of ammonia | 1 | | — | | — | | 1 | |
| | Uric acid and phosphate of lime | — | 11 | 1 | 4 | — | 3 | 1 | 18 |
| | Uric acid oxalate of lime and triple phosphate | 1 | | — | | — | | 1 | |
| | Carbonate of lime and phosphate | 2‖ | | 1 | | — | | 3 | |
| | Uric acid and oxalate of lime, with a partial coat of fusible | — | | 1 | | — | | 1 | |
| CARBONATE OF LIME, & ANIMAL MATTER ONLY | One set looks exactly like pearls, and are supposed so to be, but their history is not known | 2 | 2 | — | — | — | — | 2 | 2 |
| | | | 95 | | 39 | | 33 | 167 | 167 |

* The nucleus is placed first, and the laminæ in the order of their succession, commas being interposed between each.
† One parcel contains fourteen similar calculi.
‡ The bottle contains many similar.
§ One parcel contains three calculi.
‖ One parcel contains several, apparently from the prostate.

The proportion between the different species of calculi, with reference to the whole number, is as follows:—

*In Windmill-street collection.*

| | | |
|---|---|---|
| Uric acid, nearly . . . . | $\frac{1}{3}$ | of the whole, |
| Cystic oxide . . . . . . | $\frac{2}{95}$ | — |
| Oxalate of lime . . . . | $\frac{1}{19}$ | — |
| Phosphate, rather above . | $\frac{1}{5}$ | — |
| Alternating, rather above . | $\frac{1}{4}$ | — |
| Mixed, nearly . . . . . . | $\frac{1}{8}$ | — |

Twenty-one of the calculi were entirely free from uric acid, being almost one-fourth; a much greater proportion than is usual, as far at least as we may judge from the published accounts.

Thirty-one, or nearly one-third, contain oxalate of lime in a notable proportion.

*In Mr. Cross's collection.*

| | | |
|---|---|---|
| Uric acid, nearly . . . . | $\frac{1}{2}$ | of the whole, |
| Oxalate of lime . . . . . | $\frac{1}{39}$ | — |
| Phosphates . . . . . . | $\frac{1}{39}$ | — |
| Alternating, nearly . . . | $\frac{2}{5}$ | — |
| Mixed, about . . . . . . | $\frac{1}{10}$ | — |

Only two calculi, or about one-twentieth, were free from uric acid; and nearly one-half contain oxalate of lime, being seventeen in number.

*In the collection at Canterbury.*

| | | |
|---|---|---|
| Uric acid, nearly . . . . | $\frac{1}{3}$ | of the whole, |
| Oxalate of lime . . . . . | $\frac{1}{33}$ | — |
| Phosphates . . . . . . | $\frac{1}{33}$ | — |
| Alternating, more than . . | $\frac{1}{2}$ | — |
| Mixed . . . . . . . . . | $\frac{1}{11}$ | — |

Three calculi in this collection were found without uric acid, being just one-eleventh; and nearly one-half contained oxalate of lime, being sixteen.

In this collection is a very singular instance of a return to the oxalic deposit, after it has been suspended by the phosphatic diathesis. It is to be regretted that the particulars of the case are not known.

Taking the calculi in the three collections under one point of view, they appear thus:—

| | | |
|---|---|---|
| Uric acid, about . . . . . . | $\frac{1}{3}$, | being 56, |
| Cystic oxide, about . . . | $\frac{1}{83}$, | being 2, |
| Oxalate of lime, about . . | $\frac{1}{24}$, | being 7, |
| Phosphates, about . . . . | $\frac{2}{15}$, | being 22, |
| Alternating, rather more than | $\frac{1}{3}$, | being 60, |
| Mixed, about . . . . . . | $\frac{1}{9}$, | being 18, |
| Carbonate of lime, about . . | $\frac{1}{83}$, | being 2, |
| | | 167 |

Only twenty-six, or about one-sixth of the whole number, were free from uric acid; and sixty-four (about two-fifths) contained oxalate of lime.

By referring to the above table, it will be at once seen that the mixture of uric acid and phosphates, so far from never occurring, is, on the contrary, by no means rare; many specimens being either composed of this mixture, or containing laminæ in which it exists. Dr. Prout, however, with whom I have conversed on this point, suggests the probability of ammonia existing in combination with the uric acid in these cases.

---

